# Melatonin Administration to Pregnant Ewes for Coccidiosis Control in Their Offspring

**DOI:** 10.3390/ani13142381

**Published:** 2023-07-21

**Authors:** Efterpi Bouroutzika, Maria Giovanna Ciliberti, Mariangela Caroprese, Vaia Kantzoura, Ekaterini K. Theodosiadou, Georgios Batikas, Marios-Lazaros Michailidis, Evaggelos-Georgios Stampinas, Zafeiro Mimikou, Georgios Pantsios, Anastasios Saratsis, Irene Valasi

**Affiliations:** 1 Faculty of Veterinary Science, University of Thessaly, 43131 Karditsa, Greece; bouroutz@uth.gr (E.B.); etheodosiadou@uth.gr (E.K.T.); gbatikas@uth.gr (G.B.); mamichailidis@uth.gr (M.-L.M.); estampinas@uth.gr (E.-G.S.); 2 Department of Agriculture, Food, Natural Resources and Engineering (DAFNE), University of Foggia, 71122 Foggia, Italy; maria.ciliberti@unifg.it (M.G.C.); mariangela.caroprese@unifg.it (M.C.); 3 Veterinary Research Institute, Hellenic Agricultural Organisation Demeter, 57001 Thermi, Greece; kantzoura@elgo.gr (V.K.); pantsios2012med.life@yahoo.gr (G.P.); saratsis@elgo.gr (A.S.)

**Keywords:** ewes, pregnancy, melatonin, coccidiosis, Eimeriosis, lambs, IgG, cytokines, leucocytes, passive immunity

## Abstract

**Simple Summary:**

Coccidiosis in lambs is a vital problem of livestock management and various solutions have been proposed. Several studies, including findings from our previous work, highlighted the antioxidant and immunomodulatory role of melatonin in ewes and lambs. In this study we applied the use of melatonin implants during pregnancy in sheep as a prophylactic agent against coccidiosis in newborn lambs. The results indicated that animals that received melatonin implants demonstrated a boosted immune response against infection with pathogenic *Eimeria* spp. Also, lambs born from melatonin-treated ewes showed better humoral and cytokine immune-related response and excreted less oocysts after experimental infection with *Eimeria* spp.. Our findings indicated that melatonin can be used as an immunomodulatory agent prenatally for lowering the vulnerability of lambs to pathogens such as coccidia, and thus for keeping infection pressure down.

**Abstract:**

In livestock research, there has been a growing interest in the impact of melatonin on both health and disease conditions. The hypothesis of the present study was that melatonin treatment prenatally could support the immune competence and growth of experimentally infected lambs. This is the first study that aimed to investigate the impact of melatonin administration throughout pregnancy on immunity and oocyst excretion of pre-partum ewes and their offspring after experimental infection with *Eimeria* species. Thirty pregnant ewes were allocated into five equal groups, ΚΜ, ΚC, CM, CC, and NC, and gave birth to 47 lambs. Ewes of the KM and KC groups were orally challenged with a cocktail of *Eimeria*-sporulated oocysts (mainly consisting of *Eimeria ovinoidalis*), on day 120 of pregnancy, as well as all the lambs at the age of 5–9 days apart from those born from the NC group (environmental control). Fecal samples were collected from all ewes before infection and at parturition and from all lambs 14 times (S0–S13), before infection and during the following 8 weeks, for counting oocysts per gram of feces (OPG). Immunoglobulin (IgG) and cytokine (IL-1β, IL-6, IL-10, IFN-γ) levels were determined in ewes’ plasma collected before infection and at parturition, in lambs’ plasma at 24 and 72 h after their birth, and in colostrum samples at parturition and 72 h later. Body weight of lambs was recorded five times from birth until the age of 60 days. Accordingly, the leucogram was evaluated in blood samples collected six times within the same period. On average, IgG concentration was higher (*p* < 0.05) in the blood of KM-ewes compared to KC and CC groups and in colostrum of KM-ewes compared to other groups (*p* < 0.001). KM-lambs had greater IgG titer and IFN-γ level than the other groups (*p* < 0.05). The IL-10/ IFN-γ ratio in KM-ewes was lower than the CC group (*p* = 0.06). Overall, the growth rate of lambs did not differ among groups (*p* > 0.05). Total oocysts’ excretion in KM- and CM-lambs was reduced by 94.9% (*p* = 0.05) and 92.6% (*p* = 0.025), respectively, compared to KC-lambs, following the 3-week period after challenge, when *E. ovinoidalis* predominated in all groups. The dominant type of leucocytes was monocytes in all experimentally infected lambs, but not in NC-lambs, while overall lymphocytes were lower in KC-lambs than in NC-lambs (*p* < 0.05). Considering that almost all young indoor-reared lambs are exposed to coccidia species during their early life, melatonin treatment prenatally could suggest an alternative management tool in alleviating infection pressure.

## 1. Introduction

Several traits in domestic animals can be influenced not only during embryonic development [1] but also during perinatal and neonatal life [2]. A challenge or insult to the immune system during neonatal life could have long-term effects on physiology and immune response in adult life [2]. The control or mitigation of long-term consequences in the immune system’s vulnerability during adult life caused by prenatal or neonatal insults has raised scientific interest with various reprogramming strategies being applied on animal models. In this regard, melatonin has been proposed as a reprogramming agent, due to its pleiotropic bioactivities and its important role during pregnancy, parturition, and foetal development [3].

Currently, the positive role of melatonin administration throughout pregnancy in heat-stressed pregnant ewes on redox status, mean number and bodyweight of lambs born per ewe, as well as on milk production, has been reported [4]. Furthermore, melatonin administration prenatally may support newborns’ adaptation and survival over the first crucial days of life by modulating cytokines and suppressing oxidative stress [5]. The immunomodulatory actions of melatonin are well defined. It serves as immune-stimulant or anti-inflammatory molecule depending on the underlying condition and the generated immune response [6].

Considering the ascertained antioxidant and immunomodulatory properties of melatonin, it can be assumed that this molecule exhibits advantageous effects on pathological and clinical outcome caused by protozoan parasites. Indeed, as indicated in models other than sheep, melatonin exerted beneficial effects against a range of protozoan-induced diseases including toxoplasmosis, African trypanosomiasis, Chagas’ disease, amoebiasis, giardiasis, leishmaniasis, and malaria [7]. However, further in vivo and in vitro investigations are necessary to determine the specific mechanisms causing melatonin’s beneficial effects, including melatonin’s receptors’ role [7].

Whether melatonin administration prenatally contributes to rendering newborn lambs less vulnerable to pathogenic agents raises an issue for further investigation, given the lack of related data in the literature. Coccidiosis is a common disease in small ruminants worldwide and creates significant economic losses, mainly due to poor productivity as a result of weight loss. Infection with pathogenic *Eimeria* species, such as *E. ovinoidalis* and *E. crandallis*, in lambs can cause severe intestinal damage by destroying crypt cells and impairing nutrient absorption [8]. In indoor rearing systems, newborn lambs are infected by ingestion of sporulated *Eimeria* oocysts from the environment during the first few days of their life [9]. A progressive increase in the cumulative incidence and intensity of oocyst excretion, starting at 2–3 weeks after birth until weaning and followed thereafter by a rapid decrease, is common [9,10]. The implementation of hygienic measures to decrease infection pressure in cohabiting susceptible animals, especially indoors where oocyst accumulation is rapid, is often a challenging task. In addition, lambs born later during the lambing period, considering its prolonged duration from autumn to spring in dairy sheep production systems in the Mediterranean, demonstrate a significant tendency to get infected earlier, probably due to the higher contamination levels of the environment deriving from lambs born earlier [9]. Therefore, infection control using anticoccidial drugs is frequently deemed necessary in order to prevent clinical outbreaks and production losses [11]. However, the recent emergence of ovine *Eimeria* spp. resistance to toltrazuril highlights the urgent need for the development of alternative control strategies [12]. As opposed to the poultry industry, a notable lack of vaccine-focused approaches is evident in ruminants, with a dearth of information on the immunological mechanisms involved in ovine Eimeriosis being particularly striking. The immunity against *Eimeria* species in ruminants is mainly based on the Th1 cellular immune response, although humoral and innate responses also exist [13,14,15,16]. Whether maternal antibodies via the ingested colostrum exert anti-coccidial effects in newborn lambs seems controversial [17,18,19]. Nonetheless, given the likelihood of early exposure of indoor-reared lambs to *Eimeria* spp., the exploration of passive immunization strategies is warranted.

The interplay between melatonin and passive immunization against infectious agents in newborn lambs may give new insights into the melatonin uses in this species. Based on the previous statement, the hypothesis of the present study was that melatonin could support the immune responses of sheep and their offspring experimentally infected with *Eimeria* species. Thus, this study was designed to elucidate the effects of melatonin administration during pregnancy on humoral and cytokine immune-related responses in pre-term ewes and their offspring, as well as on clinical and parasitological parameters after experimental infection with *Eimeria* species.

## 2. Materials and Methods

### 2.1. Experimental Design

#### Animals and Treatment

Thirty ewes of cross-Karagouniko breed, aged 2–3 years, participated in this study. The animals were randomly allocated to 5 equal groups, KM (coccidia-melatonin), KC (coccidia), CM (melatonin), CC (control) and NC (environmental control). A reproductive control was applied to all ewes at the beginning of experimental procedure, followed by natural mating [4]. In the KM and CM groups, melatonin implants (dose rate: 1 implant per ewe; Regulin, Ceva, Lisbourne, France) were administered to ewes according to Bouroutzika et al. [4]. On day 120 of pregnancy, the ewes of the KM and KC groups were orally challenged with a mixture of 5 × 10^5^ *Eimeria* sporulated oocysts, consisting of 86% *E. ovinoidalis*. The group NC was similar to CC; in both, no treatment was applied. NC-ewes participated in the study for giving birth to lambs that were later used as environmental controls.

**Figure 1:**
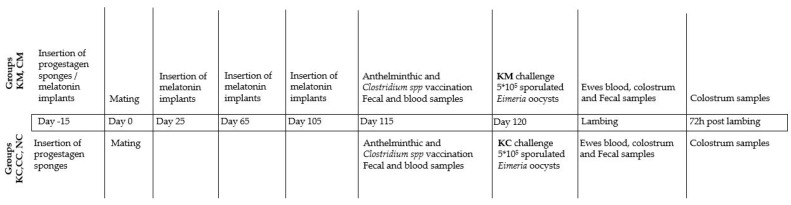
Experimental design in ewes during the study period.

**Figure 2:**
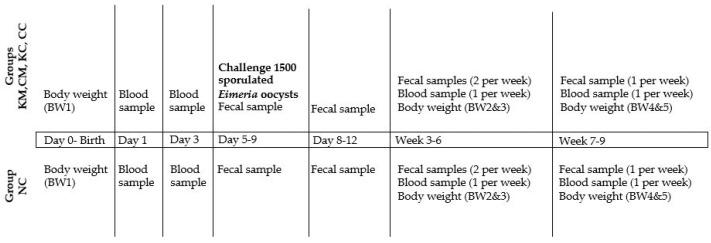
Experimental design in lambs from birth until the age of 9 weeks.

In total, 47 lambs were born; 9 from KM-ewes, 11 from KC-ewes, 10 from CM-ewes, 8 from CC-ewes, and 9 from NC-ewes, and allocated in the same groups as their dams. At the age of 5 to 9 days, the KM-, KC-, CM-, and CC-lambs were orally challenged with 1500 sporulated oocysts of the same batch. Lambs from group NC (n = 9) served as a control for the oocyst’s load in the environment. The body weight of lambs was recorded at birth (BW1) and then at the age of 14–16 (BW2), 30–33 (BW3), 45–48 (BW4), and 55–60 days (BW5). Daily monitoring of lambs was performed by a veterinarian for assessing the general health condition, including clinical signs of coccidiosis (e.g., diarrhea) throughout the study period. The detailed experimental design is described in Figure 1 and Figure 2.

### 2.2. Management Practices

The same health management practices were applied to all ewes. Specifically, after mating, ewes were fed with 300 g of ration twice a day, 1 kg clover and 2 kg alfalfa hay per ewe, and had access to water ad libitum. From the 100th day of gestation, ewes with singletons were fed with 350 g of ration twice a day, whereas ewes which bore more embryos were fed with 400 g twice a day. All ewes consumed 1.5 kg of clover and 2 kg of alfalfa hay daily and had access to water ad libitum. After lambing, the consumption of clover was increased to 1.8–2 kg per ewe daily.

On day 115 of pregnancy, anthelmintic treatment with netobimin (Hapadex^®^, Merck & Co., Inc., Rahway, NJ, USA and its affiliates) and vaccination against *Clostridium* spp. (Panclostil^®^, Ceva, Lisbourne, France) were performed. Ewes of each group were kept in separate pens, which were cleaned and disinfected before the experimental infection. As the day of lambing was approaching, ewes were placed in clean and disinfected lambing pens and after parturition were transferred into other clean and disinfected pens.

### 2.3. Sampling

Blood samples were collected from all ewes by jugular venipuncture (EDTA, BD Vacutainer^®^ Blood collection tubes, Franklin Lakes, NJ, USA) at 115 day of pregnancy and at parturition. Colostrum samples were collected at parturition and 72 h post-partum.

Also, blood samples were collected from all newborn lambs by jugular venipuncture (EDTA, BD Vacutainer^®^ Blood collection tubes, Franklin Lakes, NJ, USA) at birth and 72 h later, and then once every week, starting 7 days after the experimental infection, until weaning (in total 6 samples per lamb). Collected samples were stored at −20 °C until assayed.

Fecal samples were collected from ewes before the experimental infection (115 d of pregnancy) and at parturition. Fecal samples were also collected from lambs just before the experimental infection and 13 times thereafter within the following 8 weeks, as follows: twice per week by the 6th week and then once per week for the remaining 3 weeks (in total 14 fecal samples per lamb, S0–S13). The fecal samples were stored at +4 °C and analyzed within two days, as detailed below in 2.7.

### 2.4. Leukocyte Assay

According to Katsogiannou et al. [20] the count of white blood cells [(WBCs), neutrophils (NEU), lymphocytes (LYMPH), band neutrophils (Band NEU), monocytes (MONO), eosinophils (EOS), and basophils (BAS)] was performed using blood smears stained with Giemsa stain for each sample separately. A leukocyte differential count (200 cells) was microscopically calculated, as well as the neutrophils per lymphocytes ratio (N/L) and immature per total neutrophils ratio (I/T).

### 2.5. IgG Assay in Blood and Colostrum Samples

The IgG titer was measured in plasma samples of ewes and lambs and in colostrum samples using a specific Sheep IgG Enzyme-Linked Immunosorbent Assay (ELISA) kit (Wuhan Fine Biotech Co., Wuhan, China) as suggested by the manufacturer’s specifications. Prior to performing the analysis, the samples were diluted using Phosphate Buffer Solution (PBS) and the plates were read at 450 nm by a spectrophotometer (Power Wave XS, Biotek, Winooski, VT, USA). The intra-assay CV was 10%.

### 2.6. Cytokine Assays in Blood and Colostrum Samples

The level of IFN-γ, IL-10, IL-1β, and IL-6 cytokines was measured in ewes’ and lambs’ plasma and in colostrum samples. Colostrum samples were centrifuged at 4 °C for 20 min at 4000× *g* to separate the fat, and the supernatant was collected and diluted 1:2 in PBS.

The level of IL-10 was determined by ELISA assay as reported in Kwong et al. [21], with some modification [22]; whereas, the IFN-γ level was measured according to Ciliberti et al. [23]. In brief, 96-well plates (Sterilin, Newport, UK) were coated overnight at 4 °C with a mAb anti-bovine IL-10 and with anti-bovine IFN-γ (Serotec Ltd., Oxford, UK; 2 μg/mL), respectively, diluted in buffered carbonate (pH 9.6). After blocking non-specific binding with Bovine Serum Albumin at 3% (BSA) prepared in PBS and Tween 20 (PBST, 0.05% Tween 20), IL-10 recombinant protein (Serotec Ltd., Oxford, UK) and IFN-γ recombinant protein (Serotec Ltd., Oxford, UK) as standard in a serial dilution, serum or colostrum samples were added and incubated for 1 h. Biotinylated secondary anti-bovine IL-10 mAb and anti-bovine IFN-γ antibody (Serotec Ltd., Oxford, UK; 2 μg/mL in PBST/1% BSA) were added for 1 h. Then, the streptavidin–horseradish peroxidase (1/500 in PBS, Serotec Ltd., Oxford, UK) was added for 45 min. Finally, 3,3′, 5,5′-tetramethylbenzidine substrate solution was added for 30 min, and the colorimetric reaction was stopped by adding H_2_SO_4_ (2 M). All plates were read at 450 nm by a spectrophotometer (Power Wave XS, Biotek, Winooski, VT, USA) and data were expressed as ng/mL.

The IL-6 and IL-1β levels were performed using a sandwich ELISA, according to Ciliberti et al. [22]. Sheep antibody against IL-6 and IL-1β were used to build the sandwich. Plasma and colostrum samples were read using a standard curve obtained from serial dilution of recombinant ovine IL-6 (Cusabio Biotech Co., Wuhan, China) and recombinant bovine IL-1β diluted in PBS (Kingfisher Biotech Inc., St. Paul, MN, USA). Data were expressed as ng/mL. Plates were read at 450 nm by a spectrophotometer (Power Wave XS, Biotek, Winooski, VT, USA). All the incubations were conducted at 37 °C for 1 h, and after each step, the plates were washed 4 times. The intra-assay CV was around 10% for IFN-γ, IL-10, IL-1β, and IL-6.

### 2.7. Parasitological Examination Methods

Fecal consistency score and oocyst excretion per gram of feces (OPG) were assessed for each fecal sample. Fecal samples were scored on a scale of 1–3, where 1 indicates solid consistency, 2 pasty consistency and 3 diarrhea. The quantification of oocysts in fecal samples was performed using the modified McMaster method, where saturated sodium chloride solution plus 500 g glucose per liter was used as a flotation solution [10,11]. Additionally, *Eimeria* oocysts from samples with an OPG > 5000, provided a sufficient amount of feces was available, were sporulated in 2% potassium dichromate solution, in order to perform species differentiation [10,11]. The identification of *Eimeria* species was carried out microscopically, based on the morphology of sporulated oocysts, and 100 oocysts per sample were differentiated [12].

### 2.8. Statistical Analysis

Weight and weight gain among different groups over time were assessed by means of a repeated measures ANOVA using the package *rstatix* [13] from the R statistical language [14]. Distribution of the data and homogeneity of variances was checked by applying the Shapiro–Wilk and Box’s M tests, respectively.

IgG and cytokines data were checked for normality; when it was not satisfied a transformation was performed before analyses. A MIXED ANOVA model of SAS (SAS, 2013) was performed to compare data among treatments and time of sampling. The model included the fixed effects of the treatments (ΚΜ, ΚC, CM, and CC experimental groups) and the time (before the experimental infection (I) and at parturition (P) for ewes’ samples, at 24 h and 72 h after lambs’ birth for lambs’ samples, and at parturition (P) and 72 h after parturition for colostrum samples), and their interaction. Animals are included in the model as random effect. The significance of the differences was assessed using a Tukey post-hoc test for multiple comparisons.

The statistical analysis of white blood count (WBC) was performed using repeated measures ANOVA to assess the effect of sampling point (S2, S4, S6, S8, S10, S12), of the treatment (group) and their interaction on the various types of WBCs. Normality of data distribution was assessed with the Shapiro–Wilk test and homogeneity of variances was evaluated with the Levene test. Post-hoc comparisons were performed by means of a Tukey post-hoc test.

The area under the curve (AUC) of OPG values was used as a proxy for the total amount of oocysts excreted by each lamb during the trial (calculated for the time periods S1–S7, S8–S13 and S1–S13). The non-parametric Kruskal–Wallis test followed by a Dunn’s multiple comparison test were subsequently applied in order to compare AUC values, given that AUC values were not normally distributed across different groups.

Lamb OPG values were analyzed by means of a zero-inflated negative binomial model, due to the excess number of zeros in the dataset, using the function *mixed model* and family object *zi.negative.binomial* from package *GLMMadaptive* [15]. The model comprises two constituents: a binary model to determine the likelihood of zero counts and a negative binomial model to estimate the count distribution for non-zero counts. Model fit was improved by removing outlier values. Intervention groups and the time points S1–S13 (S) were used as fixed effects, whereas each animal was used as a random effect in the model. The NC group was set as the reference group.

Fecal score values were analyzed by means of a cumulative link mixed model using the function *clmm* in the package ordinal [16]. Intervention groups and time points S1–S13 (S) were used as fixed effects, whereas each animal was used as a random effect in the model.

Pearson correlations were calculated for multiple relationships among IgG or cytokines’ data in ewes or lambs’ blood and colostrum in each group. Also, correlations were performed among OPG, fecal score, and bodyweight in lambs. Moreover, Pearson’s correlations were performed between OPG, fecal score, neutrophils (NEU), band neutrophils (Band NEU) and monocytes (MONO) at sampling times S2, S4, S6, and S8.

In all cases, a *p* value of <0.05 was considered statistically significant, while a *p* < 0.10 was considered a tendency. Data were presented as mean ± SE.

## 3. Results

### 3.1. Lambs’ Body Weight Gain

**Figure 3:**
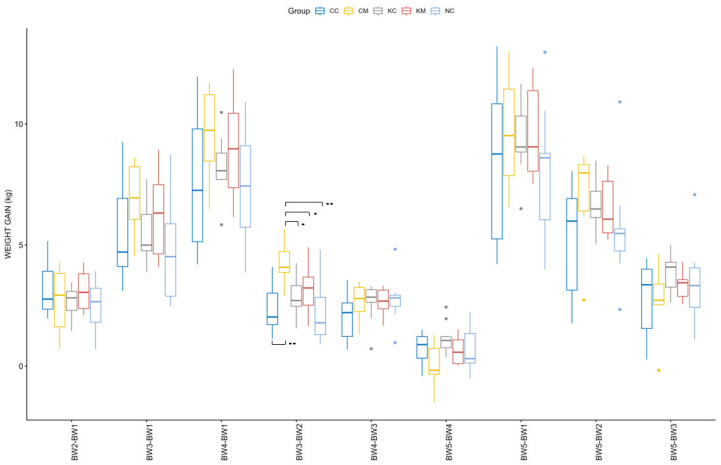
Distribution of body weight gain of lambs as calculated from weight difference between various time points (i.e., BW2-BW1, BW3-BW2, BW4-BW1, BW3-BW2, BW4-BW3, BW5-BW4, BW5-BW1, BW5-BW2, and BW5-BW3) in KM (coccidia-melatonin), KC (coccidia), CM (melatonin), CC (control) and NC (environmental control) group. Time points explanation: at birth (BW1) and on days 14–16 (BW2), 30–33 (BW3), 45–48 (BW4), and 55–60 days after birth (BW5). Significant differences across groups are marked with asterisks (* *p* ≤ 0.05, ** *p* ≤ 0.01).

Considering a normal distribution and equal variances of weight among groups, only the main effect of age [F (1.45, 53.71) = 471.983, *p* < 0.001] was found to be significant in the ANOVA repeated measures model. The effect of the treatment group [F (4.00, 37.00) = 0.654, *p* > 0.05) and its interaction with age [F (5.81, 53.71) = 1.470, *p* > 0.05] were not significant. The distribution of weight gain across different time points is depicted in Figure 3. Both age [F (8, 296) = 311.522, *p* < 0.001] and its interaction with group [F (32, 296) = 2.075, *p* < 0.001] were found to be significant. Pairwise comparisons across groups and different time points revealed a significantly higher weight gain of group CM as compared to the rest of the groups during patency of *E. ovinoidalis* experimental infection, namely between the ages of 14–16 (BW2) and 30–33 (BW3) days (Figure 3). No significant differences were observed when comparing weight gain across groups for the duration of the trial (birth to 55–60 days of age, BW1-BW5).

### 3.2. White Blood Cells Count

Mean values of total WBCs, total NEU, EOS, and N/L ratio were not significantly affected by group, neither the interaction of group with time was significant, but they were affected by the sampling time (S2–S12), (Supplementary Materials, S.M.1-Table S1, *p* < 0.05). Total BAS did not differ neither between groups, nor within time (*p* > 0.05). Mean values of LYMPH, band NEU, MONO, and I/T ratio were different between groups and were affected by the sampling time (S2–S12) (Supplementary Materials, S.M.1-Table S1, *p* < 0.05), but the interaction of group with time was not significant.

### 3.3. IgG Levels in Blood of Ewes and Lambs and in Colostrum Samples

**Figure 4:**
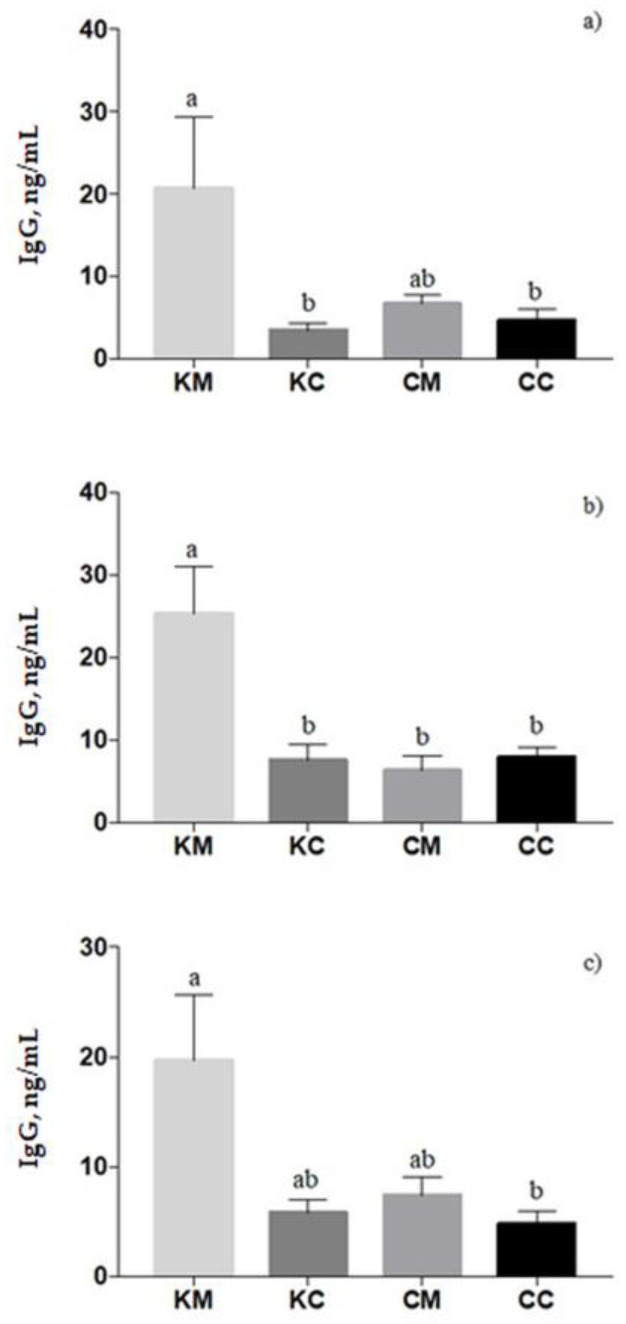
Mean of IgG titers (ng/mL ± SE) of ewes’ plasma (**a**), colostrum (**b**), and lambs’ plasma (**c**) in ΚΜ (coccidia-melatonin), ΚC (coccidia), CM (melatonin), and CC (control) experimental groups. Different superscripts (a, b) denote significant differences (*p* < 0.05).

In ewes’ plasma, the IgG titer was affected by treatment (*p* = 0.003), with a higher level in KM-ewes compared to KC- and CC-ewes (*p* < 0.05, Figure 4a). Similar results were found in colostrum samples in which a significant treatment effect was found (*p* = 0.0006); the KM group registered the highest level of IgG concentration (Figure 4b). In lambs’ plasma, the IgG concentration increased from 24 to 72 h of newborns’ life (*p* = 0.0014). Moreover, lambs born from KM-ewes showed an increase in IgG values than lambs born from CC-ewes (*p* = 0.04), on average (Figure 4c). Correlations between the IgG in blood and colostrum are presented in the Supplementary Materials (S.M.2).

### 3.4. Cytokine Secretion in the Blood of Ewes and Lambs and in Colostrum Samples

In ewes’ plasma, no significant effects were registered for IL-1β, IFN-γ level, and IL-6 cytokines (Table 1). Time significantly affected the level of IL-10, showing an increased level at pre-infection in comparison to parturition (*p* = 0.0027). The ratio of IL-10/ IFN-γ, a measure of the Th2/Th1 cytokine ratio, did not register any significant effects in ewes (*p* = 0.569 and *p* = 0.118 for treatment and time effects, respectively).

Pearson correlation analysis revealed that, in CC-ewes, the plasmatic IgG correlated with IL-6 (*p* = 0.04, r = 0.73), whereas the IFN-γ tended to correlate with the IL-1β (*p* = 0.06, r = 0.94). In CM-ewes the IgG had a correlation with IL-10 (*p* = 0.03, r = 0.68), and the IFN-γ significantly correlated with IL-10 (*p* = 0.01, r = −0.85). In KM-ewes’ plasma the IFN-γ significantly correlated with IL-1β (*p* = 0.04, r = 0.83).

The cytokines’ level in colostrum samples is presented in Table 2. On average, the level of IFN- γ, IL-1β, IL-10, and the ratio of IL-10/ IFN-γ increased significantly from parturition to 72 h postpartum (*p* = 0.01 for IFN-γ, *p* = 0.003 for IL-1β, *p* < 0.001 for IL-10, and *p* = 0.001 for the ratio IL-10/ IFN-γ, respectively). The IFN-γ level registered a tendency in the interaction effect (*p* = 0.09), in which the level of IFN-γ in the CM group at 72 h postpartum was higher than the CM group at parturition (*p* = 0.03), and the KM group at 72 h postpartum (*p* = 0.07). The level of IL-10 was significantly affected as a tendency by the interaction effect of treatment with time (*p* = 0.06); the IL-10 level in both the KM and KC groups at 72 h postpartum was higher than that registered at parturition. With regard to the IL-10/ IFN-γ ratio, a significant interaction effect was registered (*p* = 0.009), in which the KM group at 72 h postpartum had a higher ratio than the KC group at parturition.

Analysis of correlations between IgG and cytokines measured in the colostrum showed that, in the CM group, the IgG correlated with IL-10 (*p* = 0.03, r = 0.82) and IL-1β (*p* = 0.07, r = 0.71), and the IL-6 correlated with IL-10 (*p* = 0.004, r = 0.79) and IL-1β (*p* = 0.02, r = 0.58); this last showed a correlation with IL-10 (*p* < 0.001, r = 0.78). In the KC group, the IgG correlated with IL-6 (*p* = 0.05, r = 0.75), IL-1β (*p* = 0.03, r = 0.74), and IL-10 (*p* = 0.03, r = 0.79), while the IL-6 correlated with IL-1β (*p* = 0.0008, r = 0.73) and IL-10 (*p* = 0.0009, r = 0.77), and the IL-1β with IL-10 (*p* = 0.0002, r = 0.73). Finally, in the CC group, the IL-1β correlated with IL-10 (*p* = 0.001, r = 0.67), and in the KM group the colostrum IL-1β correlated with IL-10 (*p* = 0.0002, r = 0.72) and with IFN-γ (*p* = 0.01, r = −0.51).

The concentration of cytokines in the plasma of lambs is reported in Table 3. The IFN-γ concentration was affected by treatment (*p* = 0.03). On average, KM-lambs registered a higher level of IFN-γ than CM-lambs. Additionally, the level of IL-10 was affected by the interaction of treatment with time (*p* = 0.03), exhibiting a higher level of IL-10 in the KC than in the CC group at 24 h of newborns’ life. No significant effects for the level of IL-6 and IL-1β cytokine concentrations were found. The Th2/Th1 cytokine ratio was affected by treatment (*p* = 0.09) as a tendency, and the KM group had a lower ratio than CC ones (*p* = 0.06).

The highest numbers of correlations were found in the KM group, in which the colostrum IgG correlated with IL-6 (*p* = 0.007, r = 0.89) and with IFN-γ (*p* = 0.001, r = 0.89), and the lambs’ plasma IgG correlated with both IL-6, as a tendency (*p* = 0.10, r = −0.52), and with IL-1β (*p* = 0.03, r = 0.92). Finally, the IFN-γ showed a correlation with IL-6 (*p* = 0.07, r = 0.60). The remaining correlations tested for each cytokine between blood and colostrum are presented in the Supplementary Materials (S.M.3–S.M.6).

### 3.5. Oocyst Excretion and Fecal Scores from Ewes and Lambs

Ewes’ oocyst excretion did not significantly differ before infection and at parturition, within each or across treatment groups (Table 4).

**Figure 5:**
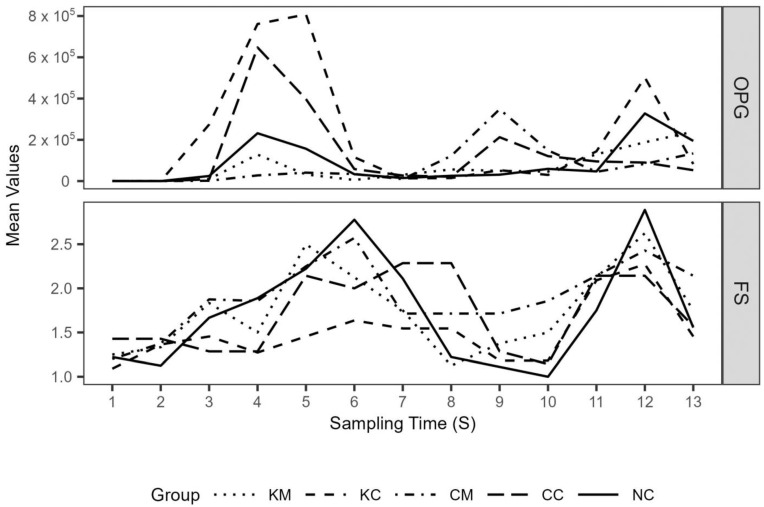
Mean oocyst excretion rates (OPG) and mean fecal score (FS) values (1, solid–3, diarrhea) in ΚΜ (coccidia-melatonin), ΚC (coccidia), CM (melatonin), CC (control), and NC (environmental control) experimental groups over the study period, which lasted 8 weeks following experimental infection of lambs. From S1 to S10, fecal samplings were performed biweekly and thereafter on a weekly basis until S13 (in total 13 fecal samples per lamb, S1–S13).

**Figure 6:**
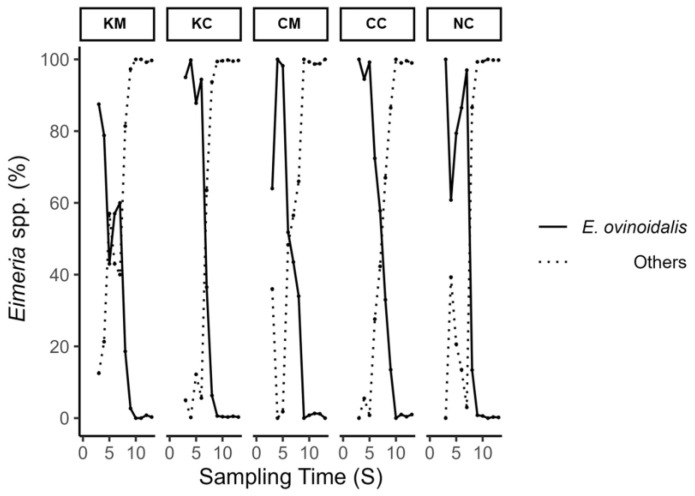
Percentage of *E. ovinoidalis* and of the remaining *Eimeria* species (others) per treatment group (KM, KC, CM, CC, NC) over the study period, which lasted 8 weeks following experimental infection of lambs (S1–S13).

Mean lambs’ oocyst excretion (OPG) and fecal score values over the study period are depicted in Figure 5. A first peak in oocyst excretion was observed on S4 and S5 (appr. 2–2.5 weeks after experimental infection), with treatment groups KM and CM consistently showing lower mean oocyst excretion values compared to the rest of the groups up to S6 and S7, respectively. This coincided with the period of *E. ovinoidalis* predominance in all groups, as after the time points S7/S8, species other than *E. ovinoidalis* predominated (Figure 6). During the study, a total of eleven *Eimeria* species were identified (*E. ovinoidalis*, *E. faurei, E. granulosa, E. crandallis/weybridgensis, E. parva, E. ahasta, E. bakuensis, E. pallida, E. marsica, and E. intricata*).

Median AUC values for the period of *E.ovinoidalis* predominance (S1–S7), a proxy for the total amount of oocysts being excreted, were 42,080, 829,480, 61,060, 134,000, and 318,780 for groups KM, KC, CM, CC, and NC, respectively. Pairwise comparisons revealed significant differences between groups KM and KC (94.9% reduction, *p* = 0.05) and between groups CM and KC (92.6% reduction, *p* = 0.025), whereas the percentage reduction of CM as compared to CC (54.5%) was not significant. Infection of ewes and their lambs (KC) without melatonin administration led to an increase in the median AUC when compared to group CC and NC; the increase was, however, not significant (*p* > 0.05). Median AUC values did not significantly differ across groups for the duration of the trial. 

The effect of treatment group (*p* < 0.05) and the interaction of the treatment group with the time points S1–S13 (S) (*p* < 0.05) were significant in the count part of the generated zero-inflated negative binomial mixed effects model. When the treatment group is considered as a single fixed effect, OPG values in KC group are expected to be 8.3 times higher (*p* < 0.01) as compared to group NC (reference group in the model), whereas OPG values in the CM group are expected to decrease (*p* < 0.05) by 91.22% [(1—exp (−2.3424) × 100] as compared to group NC, taking into account the variable time points S1–S13 (S). In the zero part of the model, no significance was observed for any of the groups.

Mean fecal score values roughly followed peaks in oocyst excretion (Figure 5b). The number of days with diarrhea (Table 5., binary variable with value 1 representing pelleted feces and values 2 and 3 diarrhea, respectively) did not significantly differ across groups for the duration of the trial (*p* > 0.05). In the generated cumulative linked mixed effects model, only the effect of time point (S) was significant (*p* < 0.05), with treatment group and its interaction with S not being significant.

### 3.6. Correlations among OPG, Fecal Score, WBC and Bodyweight

Correlations among OPG, fecal score, WBC, and bodyweight are presented in the Supplementary Materials (S.M.7–S.M.14).

## 4. Discussion

Several in vivo experimental models of infection or inflammation have reported the pleiotropic immunomodulatory actions of melatonin, but most of them evaluated the efficacy directly in the melatonin-treated animal. To our knowledge, this is the first attempt to evaluate whether melatonin administration prenatally may affect the response of lambs experimentally infected with *Eimeria* spp. from a clinical, immunological, and parasitological point of view.

It has been demonstrated that melatonin is involved in a bidirectional circuit with the immune system [6] acting as an “immunological buffer” accompanied by innate and other specific responses of the immune system. Particularly, melatonin contributes to improvement of the immune response via two main mechanisms; it amplifies antigen presentation to immunocompetent cells, enhancing the antibody response, and it modulates cytokine production resulting in controlling cellular responses [24]. Accordingly, in the present study, KM-ewes registered the higher level of IgG, both in blood and colostrum samples, compared to other groups. Another possible explanation of this result could be the positive boost effect of melatonin on the immune response, as was previously demonstrated in pregnant ewes vaccinated pre-partum against *Clostridium perfringens* [25], considering that active immune responses to infection show similarities in magnitude to vaccination response.

Colostrum uptake is a prerequisite for the survival of newborn ruminants, as it provides the passive immunity against pathogens, including coccidia, during the first days of life [19,26,27,28]. The half-life time of IgG1 in new-born lambs, the prevalent type of antibody ingested through colostrum, is approximately 11 to 13 days [16]. So, in the present study, the IgG ingested via the colostrum from lambs was present at the time of experimental infection. Thereafter, susceptibility to *E. ovinoidalis* and *E. crandallis* has been found to increase progressively in lambs up to four weeks of age [27]. Subsequently, animals acquire resistance to coccidia because of active immunity [27]. Both the general humoral immune response and the local gut humoral immune response (by IgA) are actively involved in fighting against this coccidia invasion, as was shown in goats by Matos et al. [16].

In the current study, lambs born from melatonin-treated and experimentally infected ewes (group KM) had greater titers of IgG and correlations demonstrated that the absorption of IgG via colostrum was higher than in the rest of the groups. In our previous study [5], lambs born from melatonin-treated ewes, without challenge, did not register any difference in IgG titers. However, in the latter prenatally melatonin-treated lambs, the absorption capacity gradually increased from birth to 48 h after birth, probably due to the better redox status found in the ingested colostrum. Assuming that the redox status of colostrum excreted by KM-ewes (melatonin and infection) was better than the other groups, as we have previously demonstrated in prenatally melatonin-treated lambs [5], and that the redox balance of the colostrum is closely associated with the IgG absorption, as was found in calves [29], the higher level of IgG found in KM-lambs may be attributed to a better humoral response of KM-ewes to *Eimeria* spp. challenge, which was enhanced by the antioxidant action of melatonin. Whether maternal immunoglobulins via ingested colostrum have protective anti-coccidial effects in lambs is a matter of controversy [16,17,30]. Also, as was found in calves, colostral *E. bovis*-specific antibody transfer did not result in calves’ protection [30]. Whatever the case is, the IgG levels may at least reflect the intensity of the immune response of KM-ewes to the experimental infection and the absorptive capacity of their newborn lambs, and it seems that melatonin enhanced this immune response via redox mechanism.

Furthermore, in elderly mice showing immune suppression, melatonin administration modulated cytokine production by increasing B cell proliferation and cytokines involved in Th1 response (IL-2 and IFN-γ production), and suppressing Th2 cytokines such as IL-10 [31]. Similar results are exhibited in sub-stimulated human peripheral blood mononuclear cells after in vitro melatonin treatment [32,33]. In humans, cytokine production presents diurnal rhythmicity, indicating that the IFN-γ/IL-10 peak occurs during early morning and is positively correlated with plasma melatonin [34] implying a melatonin/Th1 causality. In accordance with these statements, in our study, the lambs born from KM-ewes registered higher IFN-γ secretion in comparison with CM ones; however, they showed a higher Th2/Th1 ration than CC-ewes. These last results could be explained by the dual immunostimulant/immunosuppressive role of melatonin that has been found during immunosuppressed conditions, showing a pre-activated state useful for more effective early immune response against external stressors (i.e., viruses and parasites), as confirmed by higher IFN-γ in lambs born from challenged ewes than lambs born from mothers receiving only melatonin. Additionally, during transient or chronic exacerbated immune responses (i.e., septic shock), melatonin is considered as an anti-inflammatory molecule, promoting the Th2 response [35], as corroborated by the increased IL-10 secretion in lambs born from challenged dams (KM) in comparison with lambs born from control ones. However, when the Th2/Th1 ratio is calculated, the lambs born from challenged ewes (KM) showed a reduced Th1 ration in comparison with lambs born from a control mother. This demonstrates that melatonin could affect the modulation of immune responses in relation to the acting stressors on lambs.

Oocyst output in ewes before infection was low in all groups and this trend persisted with slightly higher, but not statistically significant, differences in mean OPG values at parturition. The inoculation of a significant number of oocysts to hyperimmunized ewes (KM and KC) did not result in any noticeable alteration in oocyst output compared to the other groups, probably due to already-established immunity. This outcome is consistent with previous research findings [18]. The predominance of *E. ovinoidalis* was observed in all lamb treatment groups (KM, KC, CM, CC) following challenge and remained consistent for a duration of two weeks (S3–S7), throughout patency. Thereafter, *E. ovinoidalis* parasite numbers gradually declined and did not reappear in significant numbers from S9 on until the end of the study, which was also previously observed [27]. This was also true for group NC (environmental control group), which demonstrated a similar course; thus, confirming an early contact with and subsequent establishment of *E. ovinoidalis*, in indoor lamb-rearing systems, where this species is prevalent [9,36].

Hyperimmunization of pregnant ewes irrespective of melatonin administration status (KM and KC), did not lead to significant weight gain compared to the respective control groups. Our findings are incongruent with prior results that indicated significant weight gain in lambs during the first month of life, which were born to hyperimmunized ewes [18]. During *E. ovinoidalis* patency, only CM-lambs gained significantly more weight, as the results from BW2 to BW3 and negative correlation between fecal score and BW2 demonstrated. Nevertheless, it is noteworthy that the remaining groups were able to compensate for this effect over the entire duration of the study. The above finding is further supported by the zero negative binomial model on oocyst excretion results, where CM-lambs are predicted to perform significantly better compared to NC-lambs, whereas hyperimmunization of pregnant ewes from the KC-lamb group is expected to result in an increased oocyst excretion compared to non-treated/non-infected control lambs (NC). Interestingly, both groups born from melatonin-treated ewes (KM and CM) excreted significantly less oocysts compared to KC-lambs during the period of *E. ovinoidalis* predominance, as expressed by AUC values. Therefore, the above results might point to a possible melatonin-mediated reduction in the susceptibility of lambs to *E. ovinoidalis* infection.

The leukogram performed in blood samples collected from lambs one week after experimental infection and thereafter, provide evidence that the innate immune response was triggered by oocysts’ ingestion. The total number of white blood cells did not differ between the five lamb groups and was within the reference values previously estimated for adult sheep [37], and lambs as well [38]. The total neutrophil number in all groups was in accordance with the lambs’ values reported by Souza et al., [38]. Interestingly, the predominant type of leucocytes, recorded in very high values compared to reference values, was monocytes in all experimentally infected lambs, but not in environmental control ones. The higher values of monocytes measured in experimentally infected lambs compared to non-infected environmental control lambs (NC) could be attributed to the ingestion of high number of oocysts; NC-lambs were obviously infected later by gradually ingesting oocysts from the environment. Monocytes/macrophages constitute one of the main cellular components of the innate response, along with neutrophils, basophils, eosinophils, and natural killer cells [6]. Macrophages created from blood monocytes act as antigen-presenting cells; they present antigens to T lymphocytes and activate them, while melatonin administration was found to enhance the antigen presentation of macrophages to T cells [39].

According to Khaksary-Mahabady [40], ovine T cells’ precursors and a concentration of T cells are present at days 44 and 55 of embryo life, respectively. Numerous studies [41,42,43] indicate that, during embryonic life, melatonin receptors are identified in the nervous system, as well as peripheral organs including the gastro-intestinal tract. Moreover, the same studies indicated that the concentration of maternal melatonin seems to have a positive impact on the number of melatonin receptors which increase during embryonic until pre-adult life. Moreover, Jimbo [44] showed that both neonatal and fetal IL-10-producing CD21+ B cells are present in jejunal Peyer’s patches, possibly for maintaining the physiological gut flora intact and safe from other enteric pathogens. In general, B and T cells and monocyte proliferation is positively affected by melatonin in inflammatory incidents [6]. Bearing all of these in mind, it can be safely assumed that a melatonin-boosted immune response at the topical level and probably reduced internal coccidia replication and, thus, oocyst excretion, are responsible for lower OPG in KM- and CM-lambs compared to the KC ones indicated. A study [45] conducted in piglets infested with *Isospora suis*, a protozoon responsible for swine coccidiosis, showed that lymphocytes were reduced in blood circulation but found in high concentrations in epithelium and lamina propria of the jejenum, implying that lymphocytes immigrate from blood to gut mucosa during active infection. This is in accordance with our results, as the lymphocytes in blood increased gradually after experimental infection in lambs, showing overall the lowest value in KC-lambs compared to NC-ones, and significantly lower values in KC-lambs compared to CM-ones, at sampling S6 (appr. three weeks after the experimental infection).

The impact of melatonin on intestinal levels should also be considered. Melatonin is also produced in high quantity by the enteroendocrine cells of intestinal mucosa, and the intestine serves both as source and recipient of melatonin produced from other sites or administered [46]. It could be assumed that in KM- and CM-lambs, melatonin treatment prenatally intensifies the intestinal immune system by activating T cells and/or protecting the intestinal mucosal barrier [47]. Studies in mice have demonstrated high affinity of the melatonin receptor (MT1) and increased number of peritoneal macrophages which produce melatonin in tryptophan-rich environments, such as the small intestine [6,48]. Furthermore, there is plenty of evidence that support the chemotactic effect of melatonin in leucocytes and the phagocytic capacity of granulocytes [6,49,50,51]. Potentially, melatonin may eliminate the invasion of sporozoites/merozoites into the enterocytes, the internal replication of parasites, and, thus, fecal oocyst excretion. This hypothesis could be further supported by the higher levels of IFN-γ found in neonate KM-lambs born from melatonin-treated ewes. IFN-γ is essential for host immunity against intracellular pathogens, as coccidia are, and it links the innate and adaptive immune system. IFN-γ increases receptor-mediated phagocytosis, respiratory burst activity, and nitric oxide production; thus, enhancing microbicidal activity of cells [52]. Also, the anti-coccidial effect of IFN-γ was revealed through reducing oocyst production in animals infected with *Eimeria* species [53]. Validation of this hypothesis remains to be ascertained by IFN-γ assay at intestinal infection sites.

The beneficial impact of melatonin administration could not only be limited to its immunomodulatory role, but also to its antioxidant properties, considering that oxidative stress is generated by coccidiosis [54]. Although, redox biomarkers were not assayed in the present study, based on our previous study where the same experimental protocol with melatonin administration was applied [5]; better redox status was found in melatonin-treated offspring, as well as higher antioxidant capacity in the colostrum of melatonin-treated ewes compared to control ones. Similar results were obtained after administration of antioxidant minerals against coccidiosis in lambs [55]. According to the latter study, lower excretion of *Eimeria* spp. oocysts and limitation of clinical signs were attributed to better activated antioxidant and immune response. In fact, melatonin acts on both innate and specific immune responses through interacting mechanisms, mostly involving cytokine modulation and redox balance.

Further studies are warranted in order to assess possible effects of melatonin administration prenatally on both innate and T cell-mediated immunity, as well as on intestinal mucosal immune responses, relating to coccidiosis in lambs, given their role in a number of protozoan parasitic infections and the lack of data in sheep.

## 5. Conclusions

This study indicated an immunomodulatory role of melatonin against coccidia infection in pre-term pregnant ewes and their newborn offspring. Lambs born from melatonin-treated ewes excreted less oocysts compared to KC-lambs, probably coming from richer in IgG and IFN-γ colostrum consumption, which is linked to passive immunity. Moreover, the cytokine profile of KM-lambs was characterized by a lower Th2/Th1 cytokine ratio than the CC ones, and stronger correlations among IgG and pro-inflammatory cytokines (IL-6, IFN-γ, and IL-1β) in colostrum and lambs’ plasma, signifying the role of melatonin in the modulation of the cytokine milieu in a dual immunostimulant/immunosuppressive effect. Of note, a significant reduction in OPG excretion was found in lambs treated with melatonin prenatally (KM and CM). Based on the fact that almost all young lambs are usually exposed and infected by *Eimeria* species during their early life, melatonin treatment prenatally could offer a feasible management practice for keeping infection pressure down and controlling clinical coccidiosis in indoor-reared lambs.

## Figures and Tables

**Table 1:** Cytokine concentrations in terms of IL-6, IFN-γ, IL-1β, and IL-10 (ng/mL ± SE) in ewes’ plasma ΚΜ (coccidia-melatonin), ΚC (coccidia), CM (melatonin), and CC (control) experimental groups before the experimental infection (I) and at parturition (*p*).

		Treatments								*p*-Value		
Cytokines, ng/mL	Time	KM		KC		CM		CC		Treatment (T)	Time (t)	T × t
			SE		SE		SE		SE			
IL-6	I	0.314	0.12	0.176	0.04	1.117	0.53	0.243	0.05	0.531	0.802	0.055
	P	0.448	0.14	0.926	0.19	0.049	0.008	0.658	0.49			
IFN-γ	I	72.548	57.24	104.215	69.78	35.946	14.34	81.042	54.12	0.745	0.773	0.543
	P	78.601	36.92	67.749	17.70	66.603	5.04	41.823	10.43			
IL-1β	I	21.762	9.06	11.614	4.71	10.037	3.71	30.135	18.44	0.876	0.543	0.926
	P	21.635	11.11	25.661	0	10.382	0	31.347	27.87			
IL-10	I	2.724	1.16	1.794	0.07	1.593	0.09	1.395	0.13	0.204	0.003	0.129
	P	1.128	0.14	1.542	0.10	0.960	0.15	1.388	0.18			
IL-10/ IFN-γ	I	0.067	0.01	0.023	0.02	0.051	0.01	0.033	0.01	0.569	0.118	0.180
ratio	P	0.026	0.01	0.030	0.01	0.015	0.01	0.043	0.01			

**Table 2:** Cytokine concentrations in terms of IL-6, IFN-γ, IL-1β, and IL-10 (ng/mL ± SE) in colostrum of ΚΜ (coccidia-melatonin), ΚC (coccidia), CM (melatonin), and CC (control) experimental groups at parturition (P) and 72 h later.

		Treatments								*p*-Value		
Cytokines, ng/mL	Time	KM		KC		CM		CC		Treatment (T)	Time (t)	T × t
			SE		SE		SE		SE			
IL-6	P	8.411	4.090	4.555	1.370	9.533	3.090	9.139	3.410	0.767	0.877	0.653
	72 h	4.388	1.040	8.345	2.610	6.527	1.300	5.824	1.560			
IFN-γ	P	242.178	22.250	278.639	55.790	228.446	30.770	231.529	32.840	0.693	0.01	0.090
	72 h	299.798	35.180	270.844	33.700	385.173	20.990	353.781	32.880			
IL-1β	P	7.549	1.540	10.016	2.299	11.468	1.770	9.530	1.510	0.121	0.003	0.896
	72 h	11.435	2.220	17.473	3.140	25.257	10.600	12.975	1.327			
IL-10	P	3.187 ^c^	4.800	1.017 ^c^	0.220	3.520 ^abc^	1.040	2.826 ^abc^	0.490	0.441	<0.001	0.061
	72 h	12.848 ^a^	1.320	8.564 ^a^	2.869	4.957 ^ab^	1.290	4.714 ^ab^	0.640			
IL-10/ IFN-γ	P	0.011	0.03	0.004 ^b^	0.0006	0.0185	0.006	0.017	0.005	0.673	0.001	0.009
ratio	72 h	0.065	0.003	0.03 a	0.008	0.0133	0.003	0.014	0.002			

Different letters (a, b, c) indicate significant differences (*p* < 0.05).

**Table 3:** Cytokine concentrations in terms of IL-6, IFN-γ, IL-1β, and IL-10 (ng/mL ± SE) in lambs’ plasma ΚΜ (coccidia-melatonin), ΚC (coccidia), CM (melatonin), and CC (control) experimental groups, at 24 h and 72 h after lambs’ birth.

		Treatments								*p*-Value		
Cytokines, ng/mL	Time	KM		KC		CM		CC		Treatment (T)	Time (t)	T × t
			SE		SE		SE		SE			
IL-6	24 h	0.544	0.257	1.383	1.308	1.480	0.603	0.122	0.115	0.103	0.655	0.638
	72 h	0.498	0.134	0.270	0.104	1.280	0.411	1.049	0.729			
IFN-γ	24 h	110.549	28.255	46.040	7.862	48.612	11.904	49.265	14.773	0.030	0.910	0.544
	72 h	85.320	26.494	43.168	6.077	41.400	10.616	61.137	8.415			
IL-1β	24 h	5.777	3.998	4.503	1.116	3.469	1.169	4.345	1.112	0.292	0.123	0.294
	72 h	3.641	0.988	1.444	0.308	2.903	1.071	4.572	1.204			
IL-10	24 h	1.736	0.409	2.203 ^a^	0.245	1.895	0.318	1.166 ^b^	0.192	0.153	0.866	0.031
	72 h	1.530	0.153	1.396	0.096	1.547	0.082	1.557	0.102			
IL-10/IFN-γ	24 h	0.019	0.0156	0.060	0.017	0.069	0.014	0.055	0.019	0.088	0.376	0.675
ratio	72 h	0.039	0.0159	0.039	0.013	0.063	0.017	0.030	0.017			

Different letters (a, b) indicate significant differences (*p* < 0.05).

**Table 4:** Mean oocyst excretion per gram of feces (mean OPG ± SE) in ewes of ΚΜ (coccidia-melatonin), ΚC (coccidia), CM (melatonin), and CC (control) experimental groups, before infection and at parturition. No significant differences (*p* > 0.05) were observed within and across groups.

OPG	KM	KC	CM	CC
Before infection	57 ± 49	63 ± 28	12 ± 12	20 ± 14
At parturition	230 ± 105	760 ± 382	360 ± 183	464 ± 377

**Table 5:** Number of days with diarrhea (fecal scores 2 and 3) and pelleted feces (fecal score 1), calculated as the percentage of all sampling points in ΚΜ (coccidia-melatonin), ΚC (coccidia), CM (melatonin), CC (control), and NC (environmental control) experimental groups over the study period S1–S13. No significant differences were observed across treatment groups (*p* > 0.05). CI: confidence interval.

Fecal Score		KM	KC	CM	CC	NC
Pelleted	Count	47	84	37	38	60
	% within Group (95% CI)	47.0% (37.3–56.7%)	58.7% (45.1–70.9%)	40.7% (22.9–57.1)	41.8% (25.1–58.9)	52.2% (35.1–67.9)
Diarrhea	Count	53	59	54	53	55
	% within Group (95% CI)	53.0% (45.6–61.4)	41.3% (35.2–49.4)	59.3% (47.8–72.2)	58.2% (46.8–71.2)	47.8% (38.2–57.8)

## Data Availability

The data presented in this study are not currently available, as they are part of PhD thesis that has not yet been completed.

## Supplementary Materials

The following supporting information can be downloaded at: https://www.mdpi.com/article/10.3390/ani13142381/s1, Table S1. Mean values (cells/μl±SE) of total white blood cells (WBCs), total neutrophils (NEU), lymphocytes (LYMPH), band neutrophils (Band NEU), monocytes (MONO), eosinophils (EOS), neutrophils per lymphocytes ratio (N/L ratio) and band neutrophils per total neutrophils ratio (I/T ratio) in KM (coccidia-melatonin), KC (coccidia), CM (melatonin), CC (control) and NC (environmental control) group after the experimental infection at sampling time S2, S4, S6, S8, S10 and S12.
